# Efficient Integer Quantization for Compressed DETR Models

**DOI:** 10.3390/e27040422

**Published:** 2025-04-13

**Authors:** Peng Liu, Congduan Li, Nanfeng Zhang, Jingfeng Yang, Li Wang

**Affiliations:** 1 The School of Electronics and Communication Engineering, Sun Yat-sen University, Shenzhen 518000, China; liup77@mail2.sysu.edu.cn; 2 Guangdong Provincial Key Laboratory of Intelligent Port Security Inspection, Huangpu Customs District, Guangzhou 510700, China; nf_zhang@126.com; 3 Guangzhou Institute of Industrial Intelligence, Guangzhou 511458, China; yangjf@gz.sia.cn; 4 College of Intelligence and Computing, Tianjin University, Beiyangyuan Campus, Tianjin 300354, China; wangli@tju.edu.cn

**Keywords:** object detection, DETR, integer-only inference, edge computing

## Abstract

The Transformer-based target detection model, DETR, has powerful feature extraction and recognition capabilities, but its high computational and storage requirements limit its deployment on resource-constrained devices. To solve this problem, we first replace the ResNet-50 backbone network in DETR with Swin-T, which realizes the unification of the backbone network with the Transformer encoder and decoder under the same Transformer processing paradigm. On this basis, we propose a quantized inference scheme based entirely on integers, which effectively serves as a data compression method for reducing memory occupation and computational complexity. Unlike previous approaches that only quantize the linear layer of DETR, we further apply integer approximation to all non-linear operational layers (e.g., Sigmoid, Softmax, LayerNorm, GELU), thus realizing the execution of the entire inference process in the integer domain. Experimental results show that our method reduces the computation and storage to 6.3% and 25% of the original model, respectively, while the average accuracy decreases by only 1.1%, which validates the effectiveness of the method as an efficient and hardware-friendly solution for target detection.

## 1. Introduction

Object detection is one of the core tasks in computer vision; it plays a crucial role in various real-world applications such as autonomous driving [1], intelligent surveillance [2], medical image analysis [3], and precision agriculture [4]. Traditional convolutional neural network (CNN)-based object detection methods have demonstrated remarkable performance on several public datasets such as COCO 2017 [5]. However, they still face challenges such as the difficulty of effectively modeling global relationships, the need for manually set hyperparameters, and significant computational and storage overhead.

At this point, the Transformer has emerged as a new paradigm, offering fresh possibilities for the implementation of object detection. With the success of Transformer models in natural language processing (NLP) [6], researchers have begun exploring their potential in computer vision tasks. DETR [7], as the first framework to directly apply Transformer to end-to-end object detection, pioneered a novel approach by reformulating the detection task as a set prediction problem. By integrating a CNN backbone for feature extraction and employing Transformer modules to model global relationships, DETR eliminates the need for manually designed components such as anchor boxes and non-maximum suppression (NMS), achieving a fully end-to-end training and inference pipeline. However, despite DETR’s impressive performance in detection accuracy and architectural simplification, its overall computational and memory overhead remains relatively high, posing certain challenges for practical deployment.

To reduce computational and memory overhead, model quantization has become an essential solution. Quantization techniques replace floating-point operations with low-bit parameters and integer arithmetic, significantly reducing computation and storage costs. Existing quantization methods can be categorized into two main types: post-training quantization (PTQ) [8] and quantization-aware training (QAT) [9]. PTQ discretizes the parameters of a floating-point model into a low-bit representation after training, which has a low computational cost but may lead to substantial accuracy loss due to the absence of training adaptation. In contrast, QAT simulates quantization errors during training, allowing the model to adapt to low-bit representations, thereby improving the accuracy of the quantized model.

**Figure 1:**
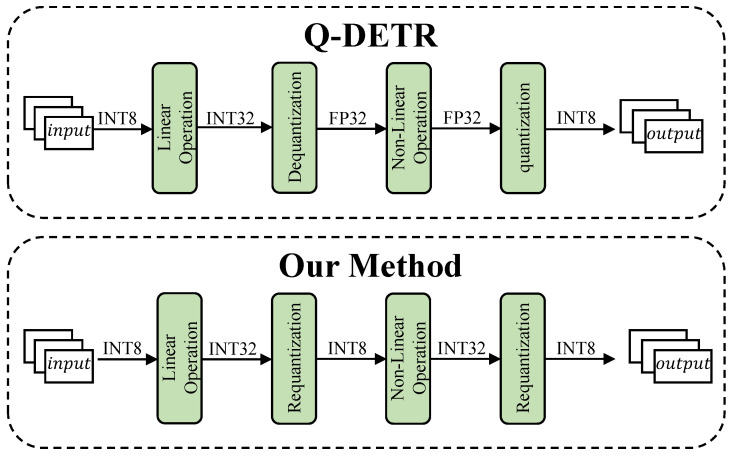
Comparison of computation flows between Q-DETR and our proposed method across different operation layers.

However, the aforementioned quantization methods primarily focus on linear operations within the model. For non-linear operations, traditional quantization approaches such as Q-DETR [10] still typically adopt a “dequantization to floating-point computation followed by quantization” strategy during inference, as illustrated in Figure 1. These operations often involve complex floating-point computations, including exponentiation, normalization, and square root, which cannot be efficiently executed on resource-constrained hardware platforms. Therefore, it is essential to approximate these non-linear functions into forms that can be directly executed within integer arithmetic units in order to establish an efficient hardware inference path. This approach helps avoid interruptions in the computation graph caused by mixed-precision execution, reduces data transfer overhead, and prevents increased hardware deployment costs due to the reliance on floating-point computation units.

To address this issue, we propose a fully integer quantized inference approach, where non-linear operations are approximated in the integer domain, eliminating the need for floating-point computations throughout the entire inference process. This allows the model to be deployed solely on integer computation units, making it highly suitable for edge and embedded devices. Compared to existing methods, the contributions of this paper are as follows:

- **Stable and accurate integer approximation for Transformer-based non-linear operations**: By replacing the ResNet-50 backbone with Swin-T, all non-linear transformations in the backbone, encoder, and decoder are under a unified Transformer architecture. This architectural unification allows non-linear approximations to focus solely on the Transformer structure, eliminating the need to simultaneously account for the CNN architecture, enabling non-linear layers involving floating-point operations, such as Softmax, LayerNorm, and GELU, to be approximately implemented in the integer domain. A stability-enhanced Sigmoid approximation is also introduced to reduce errors under high dynamic range inputs. After fully integer quantization, the average precision only slightly drops from 44.6 to 44.1, demonstrating stable performance and suitability for edge deployment.
- **Fully integer quantization optimization for linear operations**: This paper applies QAT to all linear operations for integer quantization. The use of QAT enables the model to update parameters through backpropagation, thereby learning to compensate for quantization-induced distortions and alleviating the problem of cumulative quantization errors. In addition, the low-bit integer representation significantly reduces computational complexity during inference, independent of whether QAT or PTQ is used. Compared to the non-quantized version, bitwise operations are reduced by 93.7% (from 94.2 TBLOPs to 5.9 TBLOPs).
- **Energy-efficient and hardware-friendly fully integer computation**: This paper proposes a fully integer computation scheme that achieves end-to-end integer computation through fully quantized linear operations and integer-approximated non-linear operations. Unlike existing methods, this approach relies entirely on integer computation units during inference, eliminating the need for floating-point computation units. This reduction lowers hardware manufacturing costs and decreases dependence on high-power computing architectures. Furthermore, the proposed method compresses the model storage requirement to 25% of its original size (reducing from 173.95 MB to 43.49 MB), significantly lowering the deployment threshold for edge devices.

## 2. Related Work

### 2.1. Quantization Methods for Object Detection Models

Facing the increasing demand for deploying object detection models on edge devices, quantization technology has become a crucial means to reduce computational complexity, decrease memory consumption, and enhance deployment efficiency. Currently, mainstream quantization research for object detection tasks primarily focuses on traditional convolutional neural network (CNN) detectors. These studies typically revolve around binarization or ultra-low-bit quantization (e.g., 4-bit quantization and below) of weights and activation values to achieve higher compression efficiency and faster inference speed. However, such quantization methods often limit the feature representation capability of models, leading to an increased false detection rate.

To mitigate these negative effects, the BiDet method [11] was the first to introduce the information bottleneck principle into the training process of binary detection networks. By constraining redundant information in feature maps and maximizing the mutual information between detection features and target objects, BiDet effectively reduced false detection rates and outperformed existing binarization methods on the PASCAL VOC and COCO datasets. On the other hand, in object detection tasks, direct ultra-low-bit quantization without considering the non-uniform distribution of regression parameters often results in severe degradation of localization performance. To address this issue, Reg-PTQ [12] proposed an innovative two-stage post-training quantization framework and introduced the “Filtered Global Loss Integration Calibration” mechanism, effectively overcoming the quantization bottleneck in the regression branch.

Although low-bit quantization techniques based on CNNs have gradually matured, gradient instability still limits the improvement of ultra-low-bit quantization performance. To solve this problem, the fully quantized network (FQN) [13] framework proposed a dynamic quantization range calibration and hierarchical gradient stabilization strategy. By leveraging task-adaptive quantization fine-tuning techniques, FQN achieved the first-ever stable, high-performance, fully integer-based network inference in an end-to-end manner, driving the practical application of ultra-low-bit quantization technology. Additionally, to enhance the feature representation ability of compact networks, the quantization mimic method [14] combined knowledge distillation with network quantization. This approach employed a quantized teacher network to guide the student network in optimizing the feature space, significantly improving the performance of ultra-compact CNNs in real-world detection tasks and validating the potential of distillation-quantization co-optimization.

While traditional CNN detector quantization techniques have made significant progress, research on low-bit quantization for Transformer-based detectors, which have gained widespread attention in recent years, remains relatively scarce. For DETR, Q-DETR addressed the severe distortion in query representation caused by low-bit quantization by proposing a dual-layer optimization scheme based on “Distribution Rectification Distillation” (DRD). This scheme rectifies quantized query distribution biases by maximizing self-entropy, significantly alleviating performance degradation under low-bit quantization. However, it is important to note that although this method achieved outstanding 4-bit quantization performance on the COCO dataset, it only quantized linear operations while leaving non-linear operations such as Softmax, Sigmoid, and LayerNorm not approximated. Consequently, frequent numerical type switching is required during inference, relying on floating-point computation units or hybrid computing units, making it impossible to achieve fully integer-based inference. This limitation increases hardware manufacturing costs and architecture power consumption.

These observations highlight that existing quantization schemes for Transformer-based detectors still have considerable room for optimization in practical edge device deployments. Efficiently achieving comprehensive integer optimization across both linear layers and non-linear operations remains an underexplored yet crucial research direction. This study will conduct an in-depth investigation into this key issue.

### 2.2. Model Compression and Lightweight Techniques for Transformers

Although research on the quantization of Transformer-based object detection models remains limited, significant progress has been made in lightweight Transformer explorations in other computer vision and NLP tasks. These studies provide valuable insights for optimizing Transformer models in object detection tasks.

In the field of computer vision, lightweight Transformer research mainly focuses on low-bit quantization and integer approximation optimization. For example, FQ-ViT [15] and I-ViT [16] employ integer approximation methods to optimize non-linear operations in Transformer structures (such as Softmax, LayerNorm, and GELU), enabling Transformers to maintain high inference accuracy under low-bit quantization conditions. RepQ-ViT [17] further introduces hierarchical quantization and log2 quantization, improving the stability of LayerNorm and Softmax under low-bit settings and enhancing post-quantization computational precision. Meanwhile, BiViT [18] explores extremely low-bit (binary) optimization by utilizing Softmax-aware binarization and cross-layer binarization, reducing the performance loss of Transformers under 1-bit quantization. In the natural language processing (NLP) domain, I-BERT [19] also adopts full-integer inference methods, eliminating floating-point operations in BERT computations to improve inference efficiency and reduce computational costs.

However, the application of DETR in object detection presents unique challenges. In particular, the Sigmoid layer used for bounding box regression requires adaptive modifications to better suit the object detection scenario. Based on these challenges, this study builds upon prior works and proposes an integer quantization scheme tailored for DETR. Our approach achieves full-integer computation during inference, ensuring stable detection performance after quantization. Additionally, we introduce optimized integer approximations for the Sigmoid non-linear layer, effectively addressing the accuracy loss caused by highly dynamic inputs in Sigmoid approximation computations. This enhancement improves the adaptability and robustness of DETR in object detection tasks.

## 3. Model Method

### 3.1. Model Overview

In this architecture diagram and subsequent detailed structure diagrams, we use blue-filled blocks to represent linear operation layers processed by integer quantization, and prefix such operation names with ‘Quant’, red-filled blocks to denote non-linear operation layers approximated in the integer domain, and prefix such operation names with ‘Int’. Green-filled blocks indicate composite modules that contain both linear and non-linear operations; each composite structure has been decomposed to ensure that all computational processes are ultimately implemented based on integer linear operations. Solid arrows represent the forward propagation path, and the model parameters are optimized using QAT.

**Figure 2:**
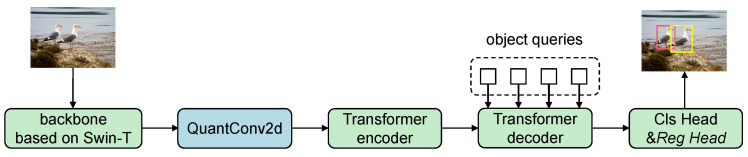
Overview of the fully quantized detection architecture.

Figure 2 illustrates the overall architecture of the proposed fully integer quantized object detection model. The model uses Swin-T as the backbone network. The extracted image features are first passed through a quantized 2D convolutional layer (QuantConv2d) to adjust channel dimensions, then fed into a quantized Transformer encoder, which captures multi-scale semantic representations. The intermediate features are then processed by a quantized Transformer decoder, which receives object queries to generate detection-aware features. These are finally passed to the classification head (Cls head) and regression head (Reg head) to output object classes and bounding box coordinates.

**Figure 3:**
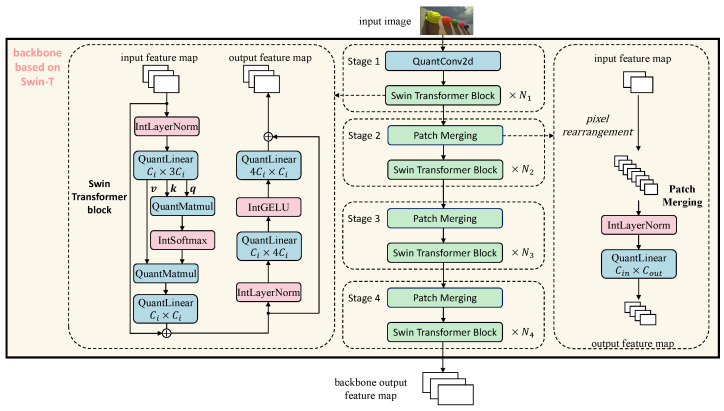
Fully integer quantized Swin-T backbone for feature extraction.

Figure 3 illustrates the fully integer-quantized Swin-T backbone architecture proposed in this work, serving as the feature extractor at the input stage of the model. The backbone is composed of four sequential stages, each responsible for progressively reducing spatial resolution, increasing channel dimensionality, and extracting hierarchical semantic features. The input image is first passed through a quantized 2D convolution layer (QuantConv2d) in Stage 1, which performs the initial spatial downsampling and mapping of the RGB image to the embedded feature dimension. Starting from Stage 2, each stage utilizes a Patch Merging operation to further reduce spatial size. Patch Merging first performs pixel rearrangement, reducing the height and width by half while increasing the number of channels fourfold. The merged features are then passed through a QuantLinear layer to project them to the appropriate dimension. The output is fed into a set of fully quantized Swin Transformer blocks, with each stage containing $N_{1},N_{2},N_{3},N_{4}$ blocks, respectively. Each Swin Transformer block is implemented entirely using integer operations, including IntLayerNorm, QuantMatmul, QuantLinear, IntSoftmax, and IntGELU. Both the self-attention mechanism and the feedforward network are structured with residual connections to ensure stable information flow. The output of Stage 4 constitutes the backbone output feature map, which is then forwarded to the Transformer-based encoder–decoder for object detection.

**Figure 4:**
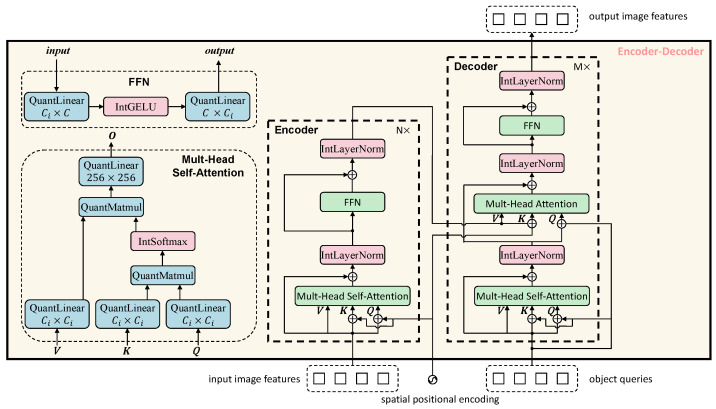
Fully Integer-quantized transformer encoder–decoder architecture.

Figure 4 presents a fully integer-quantized Transformer-based encoder–decoder architecture, consisting of an encoder on the left and a decoder on the right.

Each layer in the encoder comprises a multi-head self-attention module followed by a feedforward network (FFN), with integer-based LayerNorm and residual connections in between. Attention computations—including attention score generation, normalization via IntSoftmax, and weighted summation are all performed in the integer domain. The decoder adopts a similar layer structure but adds cross-attention modules to integrate encoded visual features with learnable object queries, enabling the model to localize and classify objects effectively. The entire model pipeline, starting from input quantization to final output generation, operates exclusively in the integer domain.

**Figure 5:**
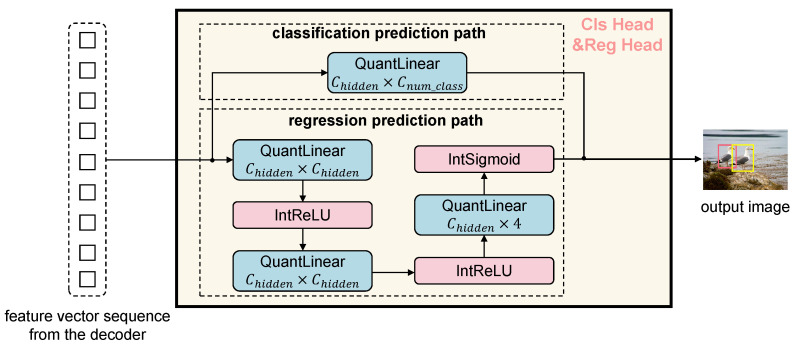
Fully integer-quantized classification and regression head.

After completing the fully integer-quantized encoder–decoder process, the output feature vector sequence from the decoder is fed into two parallel prediction heads—the Cls head and the Reg head. These two heads are also constructed entirely with integer-based operations, maintaining consistency with the fully quantized inference pipeline. This design ensures that the entire detection process remains within the integer domain, further enhancing deployment efficiency and hardware compatibility. Figure 5 illustrates the structure of the Cls head and the Reg head in the proposed fully integer-quantized object detection model. This module receives the feature vector sequence from the decoder and produces the final predictions for object categories and bounding box locations. The entire prediction head is designed following the integer inference paradigm, ensuring that all computations in the output pathway are executed entirely within the integer domain. The architecture consists of two parallel branches, namely, a classification path and a regression path.

The classification path directly maps the input features to the final number of object classes through a single QuantLinear layer, providing a lightweight and efficient classification head.

In contrast, the regression path adopts a more complex structure, comprising three fully quantized linear layers. The first two layers each include a QuantLinear operation followed by an integer activation function IntReLU for progressive feature refinement. The final QuantLinear layer outputs a four-dimensional vector representing the bounding box coordinates (x,y,w,h). Before output, an integer-based Sigmoid activation (IntSigmoid) is applied to constrain the regression values within a valid range, thereby improving the stability of location prediction.

This output structure seamlessly connects to the decoder’s output and forms a fully end-to-end integer-only detection pipeline, enhancing inference efficiency and making the model well-suited for deployment on edge devices.

### 3.2. Integer Quantization Implementation for Linear Operation Layers

After introducing the overall model architecture, we further focus on the quantization implementation of its core computational module—the linear operation layers. Since object detection models involve a large number of linear computations, applying integer quantization to these operations can significantly reduce the computational complexity and memory requirements during inference, thereby improving computational efficiency. The core idea of integer quantization is to convert model parameters and activation values from floating-point representations to low-bit integer representations, making them more suitable for efficient hardware computation. To ensure that the model maintains high performance under low-bit quantization, we have adopted a training method based on QAT. During training, our approach simulates quantization errors in advance, allowing model weights and activation values to adapt to the impact of integer quantization. Additionally, the quantized model utilizes the straight-through estimator (STE) [20] to address the non-differentiability issue caused by quantization operations during backpropagation, ensuring effective gradient propagation. In addition, all linear operation layers in this work adopt a symmetric uniform quantization strategy.

In the implementation of our model, we apply quantization to different types of linear operation layers, including fully connected layers (linear), convolutional layers (Conv2d), and matrix multiplication layers (MatMul). All these quantized layers are prefixed with “Quant” to indicate their quantized nature. Additionally, the corresponding activation quantization operations are denoted as QuantAct.

In practice, QuantAct is applied after all linear and non-linear operation layers to quantize the output activation values from Int32 to Int8, ensuring that the outputs in subsequent processing steps do not exceed the specified range. Suppose the input is *X*, and the quantization interval is $\left[-x_{\max},x_{\max}\right]$, where $x_{\max}$ is the clipping value determined by a simple min-max method. The scaling factor $S_{x}$ based on the specified number of quantization bits *k* is calculated as follows:

(1) $$S_{x}=\frac{2\cdot x_{\max}}{2^{k}-1}.$$

Next, the input data are clipped to ensure that the quantized integers during the inference phase remain within bounds. The quantized integer $I_{x}$ is as follows:

(2) $$I_{x}=\left\lfloor \frac{\mathrm{clip}(X,-x_{\max},x_{\max})}{S_{x}}\right\rceil ,$$

where $\left\lfloor \cdot \right\rceil$ is the round operator.

During the inference phase, the scaling factor $S_{x}$ determined during training remains fixed and can be converted into a dyadic number (DN) format, which translates the floating-point scaling factor into a format usable by integer computing units:

(3) $$\mathrm{DN}\left(S_{x}\right)=\frac{a}{2^{c}},$$

by performing integer multiplication with *a* and right-shifting by *c* bits, pure integer computation is achieved for linear operation layers.

The quantization of linear, Conv2D, and MatMul layers is similar to the quantization of activation values. It considers both the quantized activation values from the previous layer and the weights of the linear operation layer. For a linear layer Y=WX+b, the integer output values $I_{Y}$ and the scaling factor $S_{Y}$ are derived from the quantized integer values $I_{x}$ and scaling factor $S_{x}$ of the activation values from the previous layer, as well as the quantized integer values $I_{w}$ and scaling factor $S_{w}$ of the weights. The scaling factor for the bias is typically the product of $S_{w}$ and $S_{x}$, with the integer value denoted as $I_{b}$. Therefore, we have the following:

(4) $$Y=S_{Y}\cdot I_{Y}=WX+b=\left(S_{w}\cdot S_{x}\right)\cdot \left(I_{w}\cdot I_{x}+I_{b}\right).$$

Thus, we have the following:

(5) $$I_{Y}=\frac{S_{w}\cdot S_{x}}{S_{Y}}\left(I_{w}\cdot I_{x}+I_{b}\right).$$

By replacing $\frac{S_{w}\cdot S_{x}}{S_{Y}}$ with $S_{X}$ from Equation (3), we obtain $a^{\prime }$ and $c^{\prime }$. Therefore, we can derive the following:

(6) $$I_{Y}=\left[a^{\prime }\left(I_{w}\cdot I_{x}+I_{b}\right)\right]\gg c^{\prime },$$

where $\gg c^{\prime }$ denotes a right shift by $c^{\prime }$ bits.

### 3.3. Integer Approximation for Non-Linear Operation Layers

In the previous section, we provided an explanation of the integer quantization methods for linear operation layers and demonstrated how integer computing units can be utilized during inference to achieve efficient linear computations. However, in neural network models, non-linear activation functions and normalization operations also play a crucial role. They are essential for adjusting feature distributions, enhancing the model’s expressive capability, and stabilizing the training process. Therefore, to maintain overall computational consistency within the integer domain, it is necessary to further explore the integer approximations of non-linear operation layers.

To achieve fully integer-based inference, we propose an integer approximation scheme for non-linear layers such as ReLU, GELU, Softmax, Sigmoid, and LayerNorm. Since ReLU is linear for non-negative inputs and zero for negative inputs, approximating it only involves quantizing its non-negative portion. Later, it will be demonstrated that Sigmoid and GELU can be represented similarly to Softmax. To smooth the data distribution and prevent overflow, the exponential function in Softmax is uniformly divided by $e^{S_{x}\cdot I_{\max}}$, transforming it into the form $e^{S_{x}\cdot \left(I_{x_{i}}-I_{\max}\right)}$, where $I_{x_{i}}$ is the *i*-th quantized input and $I_{\max}$ is the maximum input value. Next, $I_{x_{i}}-I_{\max}$ is denoted as $I_{x_{i}}^{\prime }$, and the exponential function is converted into a base-2 form to facilitate integer operations and shift operations. Then the exponential function can be expressed as follows:

(7) $$e^{S_{x}\cdot I_{x_{i}}^{\prime }}=2^{\log_{2}e\cdot S_{x}\cdot I_{x_{i}}^{\prime }},$$

where $\log_{2}e$ can be approximated ${\left(1.1000\right)}_{2}-{\left(0.0001\right)}_{2}$. Therefore, we have the following:

(8) $$e^{S_{x}\cdot I_{x_{i}}^{\prime }}=2^{S_{x}\cdot I_{x_{i}}^{\prime \prime }},$$

where $I_{x_{i}}^{\prime \prime }=I_{x_{i}}^{\prime }\cdot \left(1+2^{-1}-2^{-4}\right)$.

Since $S_{x}\cdot I_{x_{i}}^{\prime \prime }$ may be non-integer and unsuitable for shift operations, we decompose it into integers *r* and *q* such that $S_{x}\cdot I_{x_{i}}^{\prime \prime }=S_{x}\cdot r+\left(-q\right)$, and satisfy $S_{x}\cdot r\in \left(-1,0\right]$. When x∈(−1,0], we have $2^{x}\approx \frac{x}{2}+1$. Therefore, we have the following:

(9) $$2^{S_{x}\cdot I_{x_{i}}^{\prime \prime }}=2^{S_{x}\cdot r}\cdot 2^{-q}\approx \left(\frac{S_{x}\cdot r}{2}+1\right)\gg q,$$

the term $\frac{S_{x}\cdot r}{2}+1$ can be rewritten as $S_{x}\cdot \left[\left(r\gg 1\right)-I_{1}\right]$, where $I_{1}=\left\lfloor -1/S_{x}\right\rfloor$.

To reduce the rounding error when right-shifting $\left[\left(r\gg 1\right)-I_{1}\right]$, left-shift the quantized integer value by *N* bits and correspondingly divide the scaling factor by $2^{N}$:

(10) $$e^{S_{x}\cdot I_{x_{i}}^{\prime }}=\frac{S_{x}}{2^{N}}\cdot \left\{\left[\left(r\gg 1\right)-I_{1}\right]\gg \left(q-N\right)\right\}=S_{\exp}\cdot I_{\exp},$$

where *N* is a predefined positive integer hyperparameter.

Thus, the Softmax can be represented as follows:

(11) $$\mathrm{Softmax}\left(S_{x}\cdot I_{x_{i}}\right)=\frac{e^{S_{x}\cdot \left(I_{x_{i}}-I_{\max}\right)}}{\sum_{j}e^{S_{x}\cdot \left(I_{x_{j}}-I_{\max}\right)}}=\frac{I_{\exp_{i}}}{\sum_{j}I_{\exp_{j}}}.$$

This integer division operation is denoted as $\mathrm{IntDiv}(I_{\exp_{i}},\sum_{i}I_{\exp_{i}},k)$, where the inputs are the dividend, divisor, and quantization bit width, respectively. The calculation process is as follows:

(12) $$\begin{array}{cc} I_{\mathrm{out}} & =\mathrm{IntDiv}\left(I_{\exp_{i}},\sum_{j}I_{\exp_{j}},k\right)\\ \; & =\left(\left\lfloor \frac{2^{M}}{\sum_{j}I_{\exp_{j}}}\right\rfloor \cdot I_{\exp_{i}}\right)\gg \left(M+1-k\right), \end{array}$$

where *M* is a sufficiently large integer. By introducing the factor $2^{M}$, truncation errors are minimized. After rounding, the value is multiplied by $I_{\exp_{i}}$ and right-shifted by (M+1−k) bits to achieve the desired target bit width. To restore the correct numerical magnitude, the corresponding scaling factor is $S_{\mathrm{out}}=1/2^{k-1}$.

Similarly, the Sigmoid non-linear operation can be expressed as follows:

(13) $$\sigma \left(S_{x}\cdot I_{x_{i}}\right)=\frac{e^{S_{x}\cdot \left(I_{x_{i}}-I_{\max}\right)}}{e^{S_{X}\cdot \left(I_{x_{i}}-I_{\max}\right)}+e^{S_{X}\cdot \left(0-I_{\max}\right)}}.$$

To maintain the numerical stability of the quantized exponential function division, we further perform the following operation on $I_{x_{i}}^{\prime \prime }$ based on Equation (8):

(14) $$I_{x_{i}}^{\prime \prime }=\max\left(I_{x_{i}}^{\prime \prime },N\times I_{1}\right).$$

In Equation (13), when the input range of $S_{x}\cdot I_{x_{i}}$ is large, the corresponding lower bound of $N\times I_{1}$ in Equation (14) increases accordingly, thus the expressions $e^{S_{x}\cdot \left(0-I_{\max}\right)}$ and $e^{S_{x}\cdot \left(I_{x_{i}}-I_{\max}\right)}$ introduce significant approximation errors due to the truncation operations. Specifically, both $I_{x}^{\prime \prime }$ and $I_{0}^{\prime \prime }$ are truncated to the same integer value $N\times I_{1}$ during the transformation process, which causes the exponential terms to become nearly identical. As a result, the output of the Sigmoid function converges to a constant value of 0.5, failing to reflect the actual variation in the input.

Since the gradient of the Sigmoid function approaches zero when the input lies outside the interval [−6,6], truncating its input has minimal impact on the final output. Furthermore, because the input undergoes Int8 symmetric uniform quantization, reducing the input range leads to a corresponding reduction in the scaling factor. This, in turn, decreases the value of $N\times I_{1}$, thereby alleviating the associated truncation error. When the input range is set to [−6,6], the corresponding scaling factor is $S_{X6}=6/127$. Meanwhile, the original input scaling factor $S_{X}$ is known both during training and inference, since it is determined by the Sigmoid input feature.

We define a non-negative integer *M* that satisfies the following condition:

(15) $$M=\left\{\begin{array}{cc} 0, & \mathrm{if}\;S_{X6}\geq S_{X};\\ \min\left\{\left(k-1\right)\in N|2^{k}\cdot S_{X6}\geq S_{X}\right\}, & \mathrm{if}\;S_{X6}<S_{X}. \end{array}\right.$$

Then, we convert the original Int8 input value $I_{X}$ of the input feature *X* into the Int32 format and apply the following transformation along with the scaling factor $S_{X}$:

(16) $$\left\{\begin{array}{cc} I_{X^{\prime }} & =\mathrm{clamp}(I_{X}\cdot 2^{M},\;-2^{7}-1,\;2^{7}-1),\\ S_{X^{\prime }} & =\frac{S_{X}}{2^{M}}. \end{array}\right.$$

After processing with Equation (16), the input range can be constrained within $\left[-x_{\max},x_{\max}\right]$, where $x_{\max}$ lies between 6 and 12. This ensures effective coverage of the non-zero gradient region of the Sigmoid function, while maintaining the scaling factor as small as possible to minimize the truncation error introduced by Equation (14).

The adjusted $S_{X^{\prime }}$ and $I_{X^{\prime }}$ are then used as the new input for the Sigmoid approximation. With these, the exponential approximations of $e^{S_{X}\cdot \left(I_{x_{i}}-I_{\max}\right)}$ and $e^{S_{X}\cdot \left(0-I_{\max}\right)}$ in Equation (13) can be implemented as integer values $I_{\exp_{x_{i}}}$ and $I_{\exp_{0}}$ via Equation (10). Accordingly, the Sigmoid function takes the following integer-approximated form:

(17) $$\sigma \left(S_{X}\cdot I_{x_{i}}\right)=\frac{I_{\exp_{x_{i}}}}{I_{\exp_{x_{i}}}+I_{\exp_{0}}}.$$

Afterward, applying Equation (12) for integer division completes the approximation process for the IntSigmoid function.

**Figure 6:**
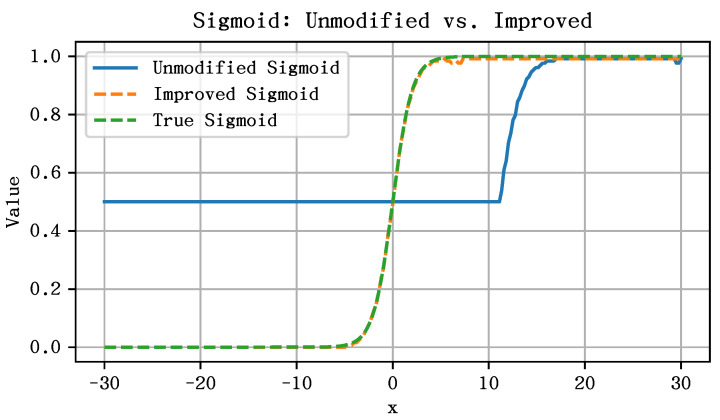
Effect of the proposed integer approximation on Sigmoid function outputs.

To validate the effectiveness of this correction, we plot the original Sigmoid approximation, the improved (corrected) Sigmoid, and the actual Sigmoid function in Figure 6. It can be observed that the improved Sigmoid function closely approximates the true Sigmoid curve.

According to [21], the GELU function can be approximately expressed as follows:

(18) GELU(X)≈X·σ(1.702·X),

where the constant 1.702 can be directly multiplied by the known input scaling factor, and the corresponding integer coefficient *a* and shift value *c* can be obtained according to Equation (3), enabling the coefficient multiplication to be implemented through bit-shifting and integer addition.

During the inference phase, the integer arithmetic unit allows LayerNorm to directly compute the mean and variance of the data but does not support the algorithm to obtain the square root of the standard deviation [22]. Solving for $\sqrt{\mathrm{var}(x)}$ is equivalent to finding the root by solving $F\left(I_{i}\right)=\mathrm{var}\left(x\right)-I_{i}$ using Newton’s iterative method:

(19) $$I_{i+1}=0.5\left(\left\lfloor \frac{\mathrm{var}(x)}{I_{i}}\right\rfloor +I_{i}\right).$$

In this case, the initial value of $I_{i}$ is set to a constant $k=2^{16}$, and this value is iteratively updated to approximate the standard deviation. In the code implementation, *k* is updated through an integer iteration process until $I_{i}$ converges to the integer approximation of the standard deviation. The number of iterations in this paper is set to 15.

## 4. Experiments

In the experimental section of this study, we sequentially investigate the impact of the backbone network on the performance of the DETR model, the optimization benefits brought by pure integer quantization compared to full-precision floating-point models, and the performance comparison between our method and other mainstream quantization approaches. The model’s recognition capability is primarily evaluated using the average precision (AP) metric on the COCO 2017 dataset.

In terms of computational complexity, following the approach in [23], we adopt bit operations (BOPs) [24] as the evaluation metric, replacing the traditional floating point operations (FLOPs). This allows for a more fine-grained quantification of the actual computational cost under different bit-width quantization schemes. The motivation for using BOPs instead of FLOPs lies in the fact that FLOPs fail to capture the variations in computational cost introduced by different bit widths. In contrast, BOPs not only provide a more accurate estimation of the computational burden in fully quantized models but also correlate closely with actual hardware energy consumption, making them a more appropriate metric for evaluating the computational complexity of quantized models. Throughout the entire experimental process, we consistently used the COCO train2017 split (comprising 118K images) as the training set and the val2017 split (with 5K images) as the test set.

Regarding the selection of the backbone network, the original version of DETR adopts ResNet-50 as its backbone. However, ResNet-50 is based on a CNN architecture and exhibits weaker feature extraction capabilities compared to Swin-T. Therefore, we replace the backbone with Swin-T, which offers stronger feature extraction ability and maintains architectural consistency with the subsequent encoder and decoder, as all components are built upon Transformer-based non-linear operation layers. This consistency facilitates the use of a unified set of integer-based approximation methods throughout the model.

To systematically analyze the impact of backbone replacement on the object detection performance of DETR, we conducted a comparative experiment between DETR models utilizing ResNet-50 and Swin-T backbones, as shown in Table 1.

The experimental results in Table 1 indicate that replacing ResNet-50 with Swin-T leads to a slight increase in computational complexity and parameter count, but also results in an improvement in the AP metric. Moreover, it facilitates the subsequent implementation of integer-approximated non-linear layers.

After selecting Swin-T as the backbone network and verifying its performance improvement on the object detection task, we further explored inference performance optimization strategies based on fully integer quantization. Although under full-precision floating-point computation, the Swin-T DETR model exhibits slightly higher computational cost and memory usage compared to the ResNet-50 DETR, the introduction of fully integer quantization significantly reduces its inference overhead, making the model more suitable for deployment on edge devices or in resource-constrained application scenarios.

To intuitively demonstrate the improvements in inference performance brought by fully integer quantization, we applied the integer-only quantization method to the Swin-T DETR model. By performing QAT based on pre-trained weights, we obtained a final quantized model and compared it with its non-quantized counterpart to comprehensively evaluate the advantages in inference efficiency. The impact of fully integer quantization on the model’s inference stage is visually presented in Table 2.

As shown in Table 2, thanks to the QAT method that effectively mitigates quantization errors and the improved integer approximation approach that better fits non-linear functions, the Int8 low-bit integer model maintains high robustness in recognition performance, with only a minor AP drop of 0.5 and an AP value of 44.1. Moreover, executing all computations in the weight, activation, and attention modules (“W/A/Attention”) using the Int8 format results in a substantial impact on both model size and bit operations. Specifically, the model storage size is reduced to only 25% of that of the full-precision model, while the number of bit operations (BOPs) drops significantly to just 6.3%, greatly reducing the inference cost.

In the aforementioned experiment, we verified the significant optimization effects of integer-only quantization in terms of computational complexity, storage requirements, and object detection accuracy. However, to more comprehensively evaluate the competitiveness of our proposed method, we need to compare it with other mainstream quantization approaches. Table 3 presents the experimental results of different quantization strategies on ResNet-50 DETR and Swin-T DETR. The upper part of the table compares the performance of ResNet-50 DETR in full precision (FP32) and under the VT-PTQ method [8], while the lower part compares the performance of Swin-T DETR in full precision (FP32) and under our proposed Integer-Only Quantization method.

The corresponding experimental results shown in Table 3 demonstrate that the proposed quantized DETR model, based on the Swin-T backbone, not only achieves fully integer-only inference but also delivers higher AP performance on object detection tasks. In addition, the quantization-induced accuracy degradation is significantly lower, which strongly verifies the effectiveness of the proposed method in maintaining model precision. These advantages facilitate efficient deployment of the model on low-power devices that support only integer arithmetic, making it better suited for real-world industrial applications where computational resources and energy consumption are strictly constrained. Therefore, the proposed method exhibits substantial practical value and strong potential for broader adoption.

**Figure 7:**
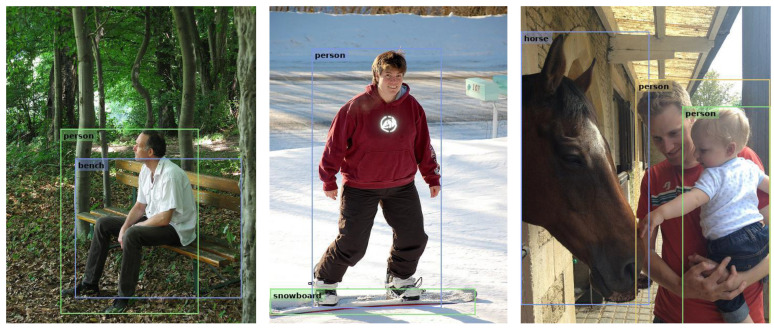
Visualization of Results from the Pure Integer Inference Model.

To more intuitively demonstrate the effectiveness of the proposed method in object detection tasks, this paper further provides visualizations of the detection results produced by the fully integer-only inference model. As shown in Figure 7, the visual results indicate that the model exhibits strong detection performance across various scenarios, including accurate localization of bounding boxes, robust detection of overlapping objects, and stable performance in complex backgrounds. This comprehensive analysis further confirms the effectiveness of the proposed model in object detection tasks.

## 5. Discussion

From an information theory perspective, the fully integer quantization-based object detection method proposed in this study is essentially an information compression and encoding optimization strategy. As a form of lossy compression, quantization replaces high-bit floating-point numbers with low-bit integers, effectively reducing model storage requirements and computational complexity. This process is analogous to signal quantization and entropy coding, aiming to preserve as much task-relevant information as possible within a limited bit-width. Specifically for Transformer architectures, our quantization scheme not only compresses linear layers but also optimizes integer approximations for non-linear operations, enabling the entire inference process to be conducted in the integer domain, thereby reducing data transmission and computational bandwidth requirements.

Moreover, the introduction of integer-based computations can be regarded as a data encoding optimization strategy. By approximating common non-linear functions such as Sigmoid, Softmax, LayerNorm, and GELU with integer-based operations, we reduce reliance on high-precision floating-point arithmetic. This is similar to optimal codebook design, where the goal is to select the most efficient numerical mappings within a constrained bit representation to maximize information retention. However, low-bit quantization inevitably introduces information loss. To mitigate this, we employ QAT to optimize errors, ensuring that the model maintains high detection accuracy even at reduced bit rates.

However, this study also has some limitations. The latest variants of DETR, such as Deformable DETR, introduce deformable attention, which significantly reduces computational complexity and improves recognition performance for deformable objects. Additionally, deformable attention enables the backbone network to output multi-layer feature maps, laying the foundation for enhanced small-object recognition performance. However, the attention mechanism in the DETR architecture scales quadratically with the input sequence length, leading to a substantial increase in computational complexity when incorporating multi-layer feature maps, which affects its feasibility. Future work will explore how to achieve an integer-based implementation of deformable attention to further enhance the model’s recognition capabilities. 

## Figures and Tables

**Table 1:** Performance comparison of different backbone networks for DETR.

Model	AP	BOPs (T)	Params
ResNet-50 DETR	42.0	88.06	41.6 M
Swin-T DETR	44.6	93.79	45.6 M

**Table 2:** Comparison of Swin-T DETR: FP32 vs. integer-only.

Bits (W/A/Attention)	AP	Size (MB)	BOPs (T)
32/32/32 (FP model)	44.6	173.95	94.2
8/8/8 (INT model)	44.1	43.49	5.9

**Table 3:** Performance comparison of DETR with different backbones and quantization methods. Bits (W-A-Attention) denotes the bit-width of weights, activations, and attention activations.

Model	Method	Bits	Size (MB)	AP	int.-only	Diff. (%)
ResNet-50 DETR	Real-valued	32-32-32	159.3	42	×	−
	VT-PTQ	8-8-8	39.8	41.2	×	−1.9
Swin-T DETR	Real-valued	32-32-32	173.9	44.6	×	−
	Our Method	8-8-8	43.5	44.1	✓	−1.1

## Data Availability

The raw data supporting the conclusions of this article will be made available by the authors upon request.

## Abbreviations

The following abbreviations are used in this manuscript:

AP	average precision
BOPs	bit operations
CNN	convolutional neural network
COCO	common objects in context
DETR	DEtection TRansformer
DN	dyadic number
FP32	32-bit floating point
FQN	fully quantized network
GELU	Gaussian error linear unit
Int8	8-bit integer
MLP	multi-layer perceptron
NLP	natural language processing
NMS	non-maximum suppression
PTQ	post-training quantization
Q-DETR	quantized detection Transformer
QAT	quantization-aware training
ReLU	rectified linear unit
Swin-T	Swin Transformer-tiny
STE	straight-through estimator
TBOPs	tera bit operations
ViT	vision Transformer
